# Diagnosis of Gonorrhœa, in Accusations of Rape

**Published:** 1842-08

**Authors:** 


*Diagnosis of Gonorrhœa, in accusations of Rape.*—The following is quoted from a recent work on the venereal disease, by Mr. Acton, late externe at the Female Venereal Hospital, Paris, of which M. Ricord is the chief medical attendant. "Every tyro in medicine will at once distinguish what he calls a clap, by means of the symptoms above described, but such a person may not be aware, that a surgeon cannot always decide at once whether a man is suffering under a gonorrhœa or not, provided no discharge be observed, and the lips of the urethra be not inflamed and no stains seen on the linen. M. Ricord gives the following instance of the occasional difficulty. He was ordered by a magistrate to give an opinion, whether or not a prisoner, said to have violated a girl, was laboring under gonorrhœa or not. The accused presented no swelling of the lips of the meatus, on pressure, no discharge came from the urethra, and there existed no traces of any secretion on the shirt. When interrogated, he said that he had made water six hours previously to his examination. As M. Ricord had some suspicion, he ordered him to pass his urine at once, and desired one of the goalers to watch his prisoner; in six hours after, M. Ricord returned and then found undoubted marks of an existing gonorrhœa; the prisoner confessed that he had made water previously to the first examination, and had taken care to remove the secretion as soon as formed by a piece of lint which he had concealed for that purpose."

The Reviewer justly doubts whether gonorrhœa can be present without an obvious vascular fulness of the mucous membrane. This should be examined with a lens. On everting the lips of the urethra, it is either seen florid, with punctuated redness, and a semi-abraded appearance, as if the epithelium were partially removed, or the veins of the mucous membrane are enlarged and tortuous.—*Am. Journ. Med. Sci.* from *Medico Chirurgical Review*, July, 1841,

